# The Aspirin Foundation Scientific Conference: the history, the present state and the future of aspirin prophylaxis

**DOI:** 10.3332/ecancer.2014.388

**Published:** 2014-01-24

**Authors:** Tom Smith, Peter Elwood, Conrad Keating, Peter Rothwell, Elmar Detering, Andrew Freedman, Ruth Langley, Richard Logan, Ceri Phillips, Andrea DeCensi

**Affiliations:** 1 The Croft, Pinwherry, Girvan, Ayrshire, Scotland KA26 0RU, UK; 2 Cardiff University, Park Pl, Cardiff CF10 3AT, Wales, UK; 3 Wellcome Institute for the History of Medicine, Oxford University, Oxford, UK; 4 Nuffield Department of Clinical Neuroscience, University of Oxford, Oxford, UK; 5 Global Medical Affairs Physician, Bayer Pharma AG 51368, Leverkusen, Germany; 6 Cardiff University and the University Hospital of Wales, Cardiff CF 14 4XW, Wales, UK; 7 MRC Clinical Trials Unit, Aviation House, 125 Kingsway, London WC2B 6NH, UK and Brighton and Sussex University Hospitals Trust, Brighton, UK; 8 University of Nottingham, University Park, Nottingham NG7 2RD, UK; 9 Swansea Centre for Health Economics, Swansea University, Singleton Park, Swansea SA2 8PP, Wales, UK; 10 Galliera Hospital, Mura delle Cappuccine 14, 16128 Genova, Italy

**Keywords:** aspirin prophylaxis, treatment, history, cost-effectiveness, vascular disease, cancer

## Abstract

The 2013 Aspirin Foundation Conference covered a range of topics from clinical and medical history, epidemiology, health economics, and the current uses of aspirin in general practice and in the treatment and prevention of cancer. The use of aspirin as primary prevention in people at risk of atherosclerotic events is now well known, but its use as a preventative agent in some cancer types is still under discussion, and data on colorectal and lung cancer were presented at this meeting. The potential use of aspirin in preventing vascular disease in HIV patients was also discussed. The cost effectiveness of aspirin as a primary prevention strategy was discussed for the first time in this series of meetings.

## Introduction

The subjects of the 2013 Aspirin Foundation Scientific Conference included clinical and medical history, epidemiology, health economics, and the current uses of aspirin in general practice and in the treatment and prevention of cancer. The talks were wide ranging and detailed, and it became clear that although aspirin is over a century old as a prescription drug, we are only now beginning to realise the full extent of its possible uses.

This report is a digest of the ten talks given at the conference, which took place, appropriately, in the house once lived in by Sir William Osler, Professor of Medicine at the University of Oxford at the turn of the 19–20th centuries. His fame remains undimmed by the century that has passed since his death, and will continue in the signs and syndromes that carry his name.

The name aspirin has survived for a similar time: it is known throughout the world. Far from being replaced by later medicines and disappearing from the world’s formularies and pharmacopoeias, it is unique amongst all of its rivals in that it is growing, rather than shrinking, in its use and indications. Why this should be and where its future lies in the prevention and treatment of diseases unrelated to its initial indication as an analgesic were the themes of this meeting.

One aspect of this future is aspirin’s role in cancer. Several speakers reported on their work with cancer in this field: the audience left much encouraged by their results. We hope that this report helps you to judge whether or not their optimism is justified.

Dr Jeffrey Aronson, Fellow of Green-Templeton College, and Reader in Clinical Pharmacology, the University of Oxford, chaired and opened the meeting. How many drugs, he asked, in the current pharmacopeia, have been used for so long as aspirin? Perhaps colchicine and digitalis, but even digitalis has lost its importance recently.

He introduced the first speaker, Dr Tom Smith, a general practitioner, medical journalist, and author, who spoke on the history of aspirin and its current use in general practice.

## Aspirin in general practice today

### Dr Tom Smith

Salicylate in the form first of willow bark has been used for more than 2000 years as an effective analgesic. Its bitter taste made it difficult to endure as a medicine until the last decade of the 19th century, Felix Hoffmann added an acetyl radical to it to form acetylsalicylic acid. It was marketed for the first time in 1899, when the common roadside weed meadowsweet (Spirea), a much more practical source of salicin than willow bark, was used as the source.

In the first decades of the 20th century, aspirin was accepted as the supreme analgesic of its time and promoted, bizarrely as we look back on it today, with the very positive claim that ‘it does not harm the heart’. It took three quarters of a century of its use solely as an analgesic and antipyretic before Sir John Vane’s work on platelets gave it a whole new life. He rightly got the Nobel Prize for his work: general prac titioners got the first medicine that would make a difference in the prevention and treatment of acute cardiovascular disease.

For general practitioners working through the last three decades of last century, the effect of aspirin was dramatic. They could give their emergency chest patients an immediate treatment that substantially improved their survival. For me and my colleagues in our rural area more than 40 miles from the nearest general hospital, at a time when there was no trained paramedical help, it was a revolution. At that time—the late 1960s to the mid-1970s—the West of Scotland was the worst place in the whole world for early death from heart disease. At that time, few of our male patients reached 75 years of age, mainly because most of them smoked. Among the over-70s in the practice, there were nine widows for each widower.

**Figure 1.**
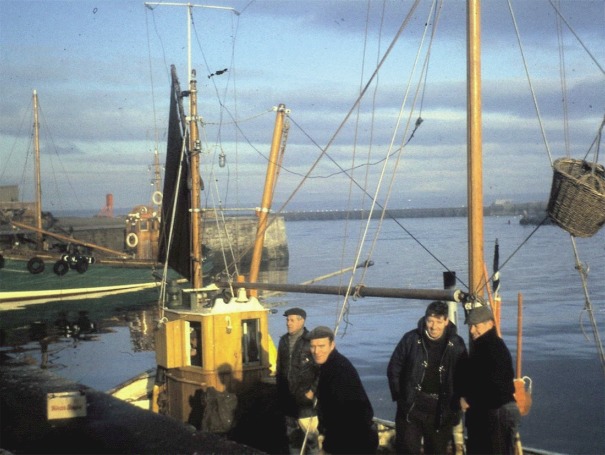
Fishing boat crew, 1968.

One example of this is in the photograph of the fishing boat crew taken in 1968 (Figure 1). Of the four crew members smiling happily in the picture, the three older men—in their forties then—all died of heart attacks in their 50s. The youngest man, then in his late 20s, survived and is still alive today, a healthy 74. The difference? In the 1970s, when the older men had their heart attacks, there was nothing the doctor could do to prevent further development of their thrombosis. They were given morphine and hope for the best. It was not the best. When Gary, the younger man, had his chest pain at age 60 in 1999, he was told by the emergency services to take an aspirin immediately. It is probable that this saved him from his colleagues’ fate.

How far have we come since the 1970s? Around a third of men over 65 in the same practice now take an aspirin every day. Life expectancy has soared among the men. Today, among our elderly care group—who meet regularly in the village hall—there are far more couples who live happily into their 80s together. The ratio of widows to widowers among the 70-year olds is near to 1:1.

Of course, the difference is not entirely due to aspirin. Far fewer men and women smoke. From a more than 70% smoking rate, now less than a quarter of our population smokes. This is thanks, partly, to Scotland being the second country in the world to ban smoking in public places in 2008. Within one year of that decision, throughout the country, our emergency admissions for heart attacks had dropped by 26%.

Whenever a chest pain call comes into our emergency services telephonists, we advise callers to take a 300-mg aspirin immediately, and to chew it until it is dissolved away in the mouth. How many lives that has saved is impossible to answer, but far more chest pain patients reach hospitals alive than ever before, even in a rural area like ours, in which ambulances can take over an hour to reach the accident and emergency department.

### Our current use of aspirin

So, what is the current use of aspirin in general practice in South-West Scotland? General practitioners are individuals, so that some may deviate from the following pattern, but for most, it is probably the commonest medication, given as 75 mg daily, for our older patients.

We offer it as prophylaxis against cardiovascular events in

(a) patients at high risk of heart attack;
(b) in complicated hypertension;
(c) in diabetes, types 1 and 2;
(d) in metabolic syndrome;
(e) in women with histories of multiple miscarriages.

In addition, it is offered after tactful discussion with selected patients, as prophylaxis against the development of cancer where there is a strong family history of colon cancer.

We give it to patients who have had surgery for colon cancer, and we are considering doing the same for patients with family histories of, or who had had, breast cancer.

Although aspirin is not yet officially approved for cancer prophylaxis or treatment, we rely on the fact that GPs retain the right to prescribe as they think, within reason. Once we have discussed the possibilities with such patients, we find that they will willingly take aspirin daily.

Of course, not everyone is enthusiastic. Many have heard that aspirin causes bleeding from the stomach. They understandably blame any attack of indigestion on the drug and stop taking it. However, a quiet and encouraging chat in the surgery usually helps. One reassurance has come from the information that a major cause of stomach bleeding on aspirin is the presence of *Helicobacter pylori*. A positive test for it leads to a course of antibiotics to eradicate it before starting aspirin prophylaxis: clinically evident gastric bleeds with aspirin are exceptionally rare after this, and they are not severe enough to be fatal.

The other worry is the possibility of an intra-cerebral bleed. The prescriber naturally worries about the relationship between aspirin and haemorrhagic stroke. Recent studies show that its incidence is much reduced if the blood pressure is kept within normal limits. So, in following-up patients on aspirin prophylaxis for whatever cause, the need to control blood pressure is paramount.

However, resistance to giving aspirin continues, sometimes among eminent physicians. In October 2013, on a primetime BBC programme, Prof Peter Elwood put forward the case for aspirin prophylaxis for most of the older men and women. To show balance, Prof Peter Sever gave the opposite view, that the risks of aspirin treatment (he stressed bleeding) outweighed its benefits, even for people at moderately increased risk of heart disease. He would not take it himself.

So, there is still an argument to be won or lost about to whom we should offer aspirin prophylaxis. The doctors who are most involved in the decision to prescribe aspirin are not the specialists or the researchers but the general practitioners. We need your help on the evidence on which we can base our decisions. We hope that the publication of this meeting will help us to do just that.

## Early trials in vascular disease

### Prof Peter Elwood

The history of aspirin in myocardial infarction (MI) and cancer starts with the history of platelets. The first to describe platelets appears to have been Albert Donne, a French physiologist, who, in 1842, described platelets as ‘globules of lymph’, which he believed coalesced into white cells. On the other hand, his contemporaries dismissed them as cell debris, or dirt on their slides. By 1874, William Osler had recognised that platelets played a part in thrombosis. He described platelet pseudopodia and the adherence of platelets to fibrin when they were stimulated.

Between 1956 and 1968, Bob Tonks and Aneuryn Hughes in Wales studied what they called platelet ‘clumping’ and Bob Tonks described the clumping as a cause of ‘infarctoid cardiopathy’. John O’Brien, a Portsmouth Haematologist, showed in 1968 that very low dose aspirin reduced platelet stickiness, and in 1974, Michael Davies proposed that plaque instability and rupture were the nidus for a platelet aggregate.

The first person to use aspirin in prophylaxis, however, did so in ignorance of all this work on platelets. Laurence Craven was an American general practitioner, and on the basis of observations about bleeding in dental work attributable to the use of aspirin for pain relief, he advised five grains of aspirin per day to 8,000 friends and patients in 1950 and published his results in the *Mississippi Valley Medical Journal* in 1956. He claimed that not a single case of cerebral or coronary thrombosis occurred among those who had faithfully adhered to the regimen during that time. Naturally, this would have been questioned today. He had no knowledge of thrombosis or of platelet function, and his papers betray the fact that he had used aspirin to reduce thrombosis for the wrong reason. He himself died of an MI in 1957.

Hughes and Tonks, on the other hand, appear to have been the first to use aspirin in vascular disease reduction for the right reasons. They worked from 1956 onwards on platelets and aspirin in ‘embolic carditis’ and ‘infarctoid cardiopathy’, producing 22 papers that are now rarely quoted and seem to have been forgotten. Having shown, with Tonks, that aspirin stabilises platelets and reduces clumps, Hughes went on in 1968 to treat patients with infarctoid cardiopathy (or MI) with aspirin. However, Dr Hughes insisted on using 5 g of aspirin a day and refused to reduce the dosage. He reported vascular reduction on a thousand patients treated with high doses of aspirin, but he abandoned the treatment when 6% of them had gastric bleeds.

The next step starts with Gasic and Gasic. Their work in 1968 in animals showed that aspirin had an antimetastatic effect in cancer, papers that were noticed by Peter Rothwell, who has gone on to research the subject in patients and is one of our speakers today. At the same time, John O’Brien stimulated the MRC to set up a randomised trial in venous thrombosis that was published in 1970. The 303 patients were given 600 mg of aspirin on the day before their operation and for five days afterwards, but it was concluded that aspirin did not even have a marginal effect on preventing venous thrombosis. That was the first randomised trial of aspirin in thrombosis.

John O’Brien wrote in *The Lancet* and elsewhere that venous thrombosis was the wrong outcome for aspirin prophylaxis and stressed that it would be more appropriate to use it in arterial thrombosis. Nevertheless, years later, a further trial in venous thrombosis with many more patients did find that aspirin did have a benefit in preventing venous thrombosis—there had been a little chance of detecting a reduction in a series of only 300 patients.

Peter Elwood took John O’Brien’s opinion seriously and decided to study arterial thrombosis. He and Archie Cochrane set up a randomised trial, based on 1,400 post-MI patients. This was published by the *British Medical Journal* under the headline ‘For Debate’. There was a 25% reduction in vascular mortality, which did not reach statistical significance. However, there was a significant reduction in vascular disease incidence, but as the original protocol had stated that conclusion would be based on mortality, this was not reported.

Over the next six years, the results of six randomised trials were published. Along with the two conducted in Britain by Cochrane and Elwood, there were one in Germany and two in the United States. Richard Peto conducted an overview of six trials and presented the results at the founding meeting of the Society for Clinical Trials in Philadelphia in 1980. There was a highly statistically significant reduction in MI on aspirin. With Richard Peto on side, there was nothing to stop the progress of aspirin, and this led to the International Study of Infarct Survival (ISIS)-2 trial set up by him and others, and eventually to the trials being presented today.

Serge Renaud also deserves a mention in this context. Renaud was highly innovative! He had worked with Frazer Mustard in McMaster University in Canada, he had given the term ‘the French paradox’ to the observation that France has a remarkably low rate of heart disease, and he had conducted a randomised trial of a diet he called ‘The Mediterranean diet’. Renaud joined Elwood’s team in Caerphilly and towed his caravan, specially equipped for platelet tests, to Caerphilly, where it was parked for eight years!

Together with Renaud, Elwood’s group did platelet tests, using three tests of aggregation, on the 2,500 men in the Caerphilly cohort. Longterm follow-up gave no evidence whatever that any aspect of platelet function was predictive of MI. On the other hand, the men with the least active platelets at baseline experienced an increased incidence in ischaemic stroke!

## 

References1.DonnéADe l’origine des globules du sang, deleur mode de formation et de leur finCompt Rend Acad Sci18421436682.OslerWAn account of certain organisms occurring in the liquor sanguinisProc Roy Soc Lond187456927343.HughesATonksRSExperimental embolic carditisJ Pathol Bacteriol19567249750310.1002/path.17007202144.HughesATonksRSMagnesium, adenosine diphosphate and plateletsNature (Lond)1966210106710.1038/210106a059563425.DaviesHTPHughesATonksRSExperimental and clinical lung and heart lesions resulting from intravascular platelet clumping and some factors in their preventionProc. of the 3rd International Pharmacological Meeting1966(S Paulo, Brazil) in Drugs in relation to blood coagulation, haemostasis and thrombosis. 1968BrazilS Paulo6.O’BrienJREffects of salicylates on platelet stickinessLancet19682917798310.1016/S0140-6736(68)92228-941711297.Steering Committee MRCEffect of aspirin on postoperative venous thrombosis. Report of a steering committee of a trial sponsored by the Medical Research CouncilLancet1972300441510.1016/S0140-6736(72)91849-141153408.ElwoodPCA randomised, controlled trial of acetyl salicylic acid in the secondary prevention of mortality from myocardial infarctionBrit Med J197459054364010.1136/bmj.1.5905.4364593555PMC16332469.PetoRAspirin after myocardial infarction EditorialLancet1980i11723610399010.ElwoodPCPlatelet aggregation and bleeding time predict stroke but not myocardial infarction: the Caerphilly CohortPlatelets20021333311.RenaudSCAlcohol and platelet aggregation: the Caerphilly prospective heart disease studyAm J Clin Nutr199255101217157079510.1093/ajcn/55.5.1012

## ISIS-2 and early overviews

### Conrad Keating, Writer-in-Residence, Wellcome Institute for the History of Medicine, Oxford University

In the William Dunn School, near where Howard Florey forged his career, on 25 May 1940, eight mice were injected with a lethal dose of streptococci. Four of the mice were treated with penicillin and four used as controls. Few drugs have had such obvious and efficacious results as the world’s first antibiotic. Howard Florey was famous for being allergic to statistics—he used to say that all his patients were either all dead or all alive!

The days of finding another drug with the dramatic effects of penicillin are far distant. The spectacular success of antibiotics, including streptomycin to treat tuberculosis had led doctors to expect that a new drug’s effectiveness would become obvious after treating only a few dozen or perhaps a hundred patients. With treatment for cancer and heart disease, the principal causes of death in the developed world it was unrealistic to expect such striking effects. Austin Bradford Hill introduced randomisation into medicine with his trial of streptomycin in the 1940s and it was such a good drug that doctors rapidly adopted it.

Randomisation comes into its own when there is no reliable consensus about a treatment. In 1969, Richard Doll came to Oxford as Regius Professor of Medicine and it transformed medical statistics and its impact on clinical medicine. As Bradford Hill’s most distinguished collaborator, he continued to work on the hazards of smoking. He attracted a new generation of researchers who had a passion for numbers and were as dedicated as he was in the need to prevent premature death.

Richard Peto joined Doll in 1967 and he believed that proving the effectiveness of drugs in preventing deaths from heart attacks was an essential part of medicine. For something as common as heart disease, a drug that was effective in only a few per cent of cases would still save many thousands of lives. To measure such modest effects reliably, drugs would have to be tested in many thousands, and not just hundreds, of patients.

Doll and Peto went for big killers. Doll was the greatest influence on reducing deaths from cancer in the 20th century, and Peto carried on this tradition in heart disease. In the 1960s and 1970s, many of the trials aimed to disprove benefit—to show that treatments did not work, rather than to show that they did. There was a strong nihilist tradition. Researchers were less embarrassed about missing a positive result than a negative one. The big trials of the 1980s were to change that nihilist attitude.

For Richard Peto and his statistically minded clinicians, the area that offered the greatest potential for saving lives was in acute MI. In 1976, Salim Yusuf came to Oxford as a Rhodes Scholar, to work with Peto and was assigned to develop a method to see if beta-blockers might reduce complications and infarct size. This study was rolled out as ISIS-1 and its distinctive feature was its simplicity. The trial was designed to have 6,000 individuals with four or five data points. The information could have been collected and put on the back of an envelope. In addition, the discharge data were on one side of a page. It was a simple trial, open, and had no control.

The British Heart Foundation supported the study and when Rory Collins arrived in Oxford 1981, he joined the team. By the time he arrived, ISIS-1 may have lost its initial steam, but it was important because it showed that a big trial could be simple and relatively cheap, and it was possible to recruit large numbers of subjects.

It was historically important because it was the first in a series of clinically important trials that dramatically altered research methods and clinical practice. It had enrolled 16,000 patients and had demonstrated reduction in MI, cardiac arrest, and death. The ISIS-1 trial results were not decisive enough, however, to have much clinical impact, but it showed how to recruit large numbers and inspired the Oxford team to undertake a much more audacious undertaking.

Rory Collins, Salim Yusuf, and Richard Peto carried out a meta-analysis of the data and ‘dug out’ the data on streptokinase and reanalysed them. Taken separately, they showed no effect, but taken together there was a 20% reduction in MI deaths in those who were allocated streptokinase.

This was the encouragement they needed to embark on their own massive trial, ISIS-2. In addition, they studied all the historical evidence on aspirin, which led them to include an aspirin arm. They added an epidemiological aspect to the trial plan—how much the subjects drank and smoked.

There were objections to the trial. First, physicians were worried about the use of streptokinase: one patient in a thousand had a bleed into their brain, so they had a right to be suspicious. The hazards were immediate and obvious. If the drug killed someone, you could see this immediately, but if it was saving lives you could not see it. This led to a very strong prejudice in many doctors’ minds against streptokinase and also against aspirin. Richard Peto said:

‘I was told by one expert that I might as well turn my bum to the wind and fart to try to stop the wind blowing as use aspirin in the middle of acute MI and so he wouldn’t randomise—because he was certain that it would not work. He also thought that streptokinase was going to be dangerous. When the results came out he instantly changed his practice and adopted both—and he also pointed out that he had been wrong’.

The feeling was that ISIS-2 might be of statistical interest, but not of clinical interest, but there had been a softening up. The meta-analysis of the streptokinase data had shown benefit and some benefits had been shown in the GISSI trial. This Italian trial had been in ISIS-1 but the researchers had dropped out of it (with the encouragement of Richard Peto) to set up a separate trial on streptokinase. GISSI was published slightly before ISIS-2 and the net effect was to increase recruitment into ISIS-2.

When GISSI was published the editorial that commented on the results stated that they were not clear and more was needed. However, it did a lot to dispel clinicians’ negative attitudes to trials. Trialists were no longer seen as boffins and nerds but as people who could provide valuable knowledge on the use of drugs in clinical practice.

ISIS-1 had cost one million pounds, and ISIS-2 was going to cost two million pounds—who was going to pay for ISIS-2? Having persuaded themselves that streptokinase should work and that aspirin might work, Richard Peto and Peter Sleight approached a German company Behringwerke, who had been making streptokinase for years, for sponsorship. Over dinner at the chairman’s house the agreement to supply the drugs and placebo plus the two million pounds was made. At the time, Behringwerke agreed to factor in aspirin despite the fact that their streptokinase packaging carried a warning not to combine the two drugs. Within a short time, the company’s accountant challenged Richard Peto with the fact that the Oxford Group was doing three trials (with streptokinase, aspirin, and an epidemiological one) and that their drug was still not in patent. They tried to buy the signature back, but Richard Peto refused, and continued with the trial.

Doctors in 417 hospitals and in 16 countries agreed to participate in ISIS-2: they amassed almost 18,000 patients. The trial was of intravenous streptokinase, oral aspirin both or neither in patients with suspected MI. The cardiologists were blind to the treatment. Fostering their cooperation was probably Rory Collins’ greatest achievement. The idea was to enrol people as quickly as possible. There was minimal administration—people could phone up. When ISIS-2 was published, all of the collaborators’ names were listed in *The Lancet*.

The ISIS-2 trial took three years. Much had been learned from the ISIS-1 trial, in that speed was of the essence. However, even with the two million pounds, margins were extremely tight and accommodation was in such short supply that at one time this huge trial was conducted in a small room appropriated (without paying rent) from the university. They were not supposed to be there and were almost evicted, but were there for three years.

As an example of the problems, when the trial was under way, Rory Collins was summoned by one physician to a coronary care ward to see a patient with extreme back pain. He was told that he was ‘killing my patient’. Fortunately, when the code was unblinded, the patient was in the placebo arm! This would have been a ‘tight’ moment according to Prof Collins, if he had been on one of the active drugs.

The results, published in *The Lancet* on 13 August 1988, were unequivocal. Streptokinase injected as soon as possible after a myocardial infarct reduced the chance of dying by more than 20%. Taking an aspirin every day had a similar beneficial outcome. When both drugs were given, the reduction was 40%. *The Lancet* comment was uncharacteristically optimistic. It stated that if both are used widely, this should avoid several tens of thousands of deaths each year.

Richard Doll was the Chairman of the Trial Data Monitoring Committee, and when they published a preliminary report in 1987, it had minimal impact. This changed with the publication of the full report in 1988. When Rory and Desmond Julian, who was the Clinical Director of the British Heart Foundation, wrote to all the cardiologists in 1987 before the results were published about their use of ‘clot-busting therapy’ for acute heart attacks their responses showed that only 2% used them routinely. By 1989, after publication, this had risen to 68% (Table 1).

It is clear that the publication of ISIS-2 in August 1988 had an immediate impact. It brought clarity out of the chaos that surrounded acute MI treatment and provided the first evidence that aspirin was beneficial in acute MI.

The big clinical question for medical researchers today is: ‘What has been the impact of your work, and how has it been translated into mortality and morbidity gained?’

With the reduction of mortality in ISIS-2, everyone knew that the effect was real and there was no need to get into complex statistical analyses. Any doctor looking at the 1,000 versus 800 deaths knew this was a real reduction—that would not have been so convincing if the figures had been ten against eight or even 100 against 80. If there had been a marginal result, clinicians would not have changed their practice. In a trial of a new therapy, one needs to blow previous hypotheses out of the water, and that is what ISIS-2 did.

It changed people’s thinking about clot busters, aspirin, and trials. It did so not only in the west but across the world. For the first time, people understood that a treatment effect, though moderate (40% is thought as moderate) was there. Before the ISIS-2 trial, doctors wanted to see big effects, like those found by Florey with penicillin. If there were no big effects, they did not accept the results.

ISIS-2 changed that need. Rory Collins draws an intriguing analogy: ‘It is like a microscope: big trials reveal what isn’t visible—you’re able to see something you didn’t know exists and you’re able to understand that it is important.’

ISIS-2 established a tradition that large-scale randomised evidence could be exciting and relevant. It also showed that there is no substitute for numbers. Statisticians do not want big numbers because they think all patients are the same, but because they know patients are different, and it is only by using them you can average out the differences. Salim Yusuf places the ISIS-2 study in cardiovascular history: ‘ISIS-2 is probably the biggest single step in the advancement of acute myocardial infarction. (And) …the ISIS trials had as big an impact on the evaluation of therapies in many diseases as did the original TB trials that Austin Bradford Hill conducted.’

One incontrovertible fact about ISIS-2 is that it could not have happened without the support and participation of Peter Sleight, who is here today. Cardiologists needed to know that they were collaborating with cardiologists and not just with boffins and nerds. Salim and Rory are clinicians, but Peter Sleight is a cardiologist’s cardiologist. As a result, his colleagues could agree to take part in something that was being run by him. He was committed to get proper evidence while he trusted Collins and Peto to run the trial and to interpret the results.

Twenty five years after the publication of the results, we know that the best way to avoid deaths in the early phase of most of the heart attacks is still based on two fundamental things—to get rid of the clot and to stop the clot from forming.

Did ISIS-2 become a template for trials? It should have done, and in many ways it did. It had a profound effect on cardiology, and cardiologists started to do their own big trials. In terms of evidence-based medicine, they are showing the way forward. However, Salim, Rory and Richard Peto all agree that the avalanche of trial regulations has stifled progress. Large-scale evidence needs big trials, so that they should be getting simpler. However, what has happened is that trials are getting smaller, more expensive, and more complex. There was an opportunity for large trials and it is disheartening to see the regression in clinical trial methodology and the increasing complexity of the conduct of trials with no real benefit in terms of protecting patients or producing more reliable results. It is an optimistic story, but with a slightly pessimistic ending.

## Early cancer studies

### Prof Peter Rothwell, Action Research Professor of Neurology, Nuffield Department of Clinical Neuroscience, University of Oxford

There are two aspects of aspirin in cancer—the short-term effect in preventing metastasis and the long-term effect on cancer incidence. It is difficult to tease them apart: they may have separate mechanisms. The 1968 studies by Gasic and Gasic in rats showed that if you get rid of the platelets in rats, you cannot seed metastases. If you inject cancer cells into veins, and platelets are there, the cancer cells ‘seed’ into the organs, causing metastases. If you artificially remove the platelets, this seeding does not take place, suggesting that the platelets play a part in this process.

They then showed that aspirin had a similar effect in their rat model. This was not seized upon at the time in the way one might think it should have been. There have been about 2,000 subsequent papers on the suggested mechanisms by which aspirin might affect cancer growth and cancer metastasis, so it has not been ignored by basic scientists. There has also been some observational works looking at patients with cancer who took or did not take aspirin, looking for effects on cancer growth. There was some observational evidences that aspirin might have prevented metastasis, tending to support the findings of Gasic and Gasic of 40 years before.

Prof Rothwell and his collaborators were interested in these reports, and wondered if they were linked to a slightly odd finding in the cardiovascular trials that Rothwell had made, namely that aspirin reduced non-vascular deaths compared with the placebo group. This was a hint that aspirin might be doing something to cancer even in the short term—the trials were mainly over two to seven years. In the primary prevention trials of aspirin, for example, there was no effect on vascular deaths but there was a fairly consistent 12% reduction in nonvascular deaths. The commonest cause of non-vascular death was cancer.

They therefore gathered the individual patient data on cancer deaths in all 51 trials of daily aspirin versus control in primary or secondary prevention of vascular disease and looked for any effect of aspirin by period of follow-up. They found that after five years, there were significantly fewer cancer deaths in the aspirin than in the placebo groups. A delayed effect was plausible if aspirin was preventing the growth or metastasis of cancers that were occult at the time that patients were randomised in these trials but that subsequently presented during follow-up.

Prof Rothwell’s group therefore looked in more detail at the five large UK trials of aspirin versus control in prevention of vascular events in which there were old paper records that should contain data on cancer incidence and behaviour during the trials. They looked at whether cancers were metastatic on presentation, became metastatic on follow-up, or whether they were localised and stayed localised, were surgically cured or were the cause of death.

Aspirin was found to have a statistically robust effect on definite distant metastasis, so that if cancer presented on aspirin or placebo, it seemed to behave differently, with significantly fewer metastatic cancers on aspirin than on placebo. Virtually all these cancers must have been developing prior to randomisation: their latent period is quite long. Looking at metastasis by site there fewer metastases to lung, liver, and brain on aspirin than on placebo, although there was no apparent effect on bone metastases—these were predominantly in men with prostate cancer, metastasising to the lumbar spine. There was also significantly better survival after diagnosis of cancer in the group randomised to aspirin than to placebo in these trials. These data come from randomised controlled blinded trials, but people were randomised before they knew they had a cancer. Current trials such as add aspirin are now looking at whether aspirin helps after diagnosis.

**Figure 2.**
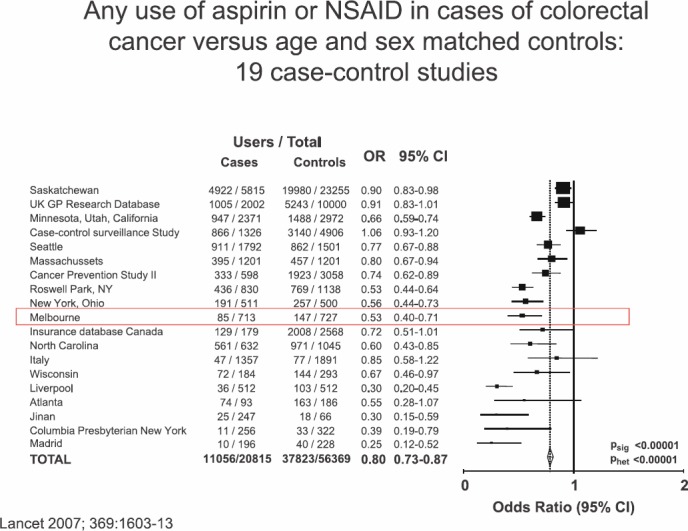
Any use of aspirin or NSAID in cases of colorectal cancer versus age and sex-matched controls: 19 case-control studies.

The second issue, separate from the short-term effects of aspirin on short-term risk of cancer and cancer metastasis, is the possible effects of aspirin on long-term cancer incidence. The first aspect of this story is the long-term effect of aspirin on incidence of colorectal cancer, which goes back to the 1980s with the Kune *et al*’s study in Melbourne. This was a traditional case-control study looking at many different factors in colorectal cancer cases versus controls, which just happened to ask ‘did the patient take regular aspirin?’ The researchers found to their surprise that aspirin use was less common in cases of colorectal cancer in controls. They hypothesised that taking aspirin might prevent colorectal cancer, and their findings were soon replicated by Michael Thun’s cohort study in Boston and subsequently by many other case control (Figure 2) and cohort studies.

There followed a series of randomised trials of aspirin or COX inhibitors in secondary prevention in patients with previous polyps or colorectal cancer (four RCTs using aspirin and three using COX-2 inhibitors). These showed a 20–30% reduction in recurrent polyps in the aspirin groups, but follow-up was too short (two to three years) to allow any effect on risk of cancer to be determined. Although up to 40% of people aged 60 have adenomas only 10% of adenomas progress to cancer. Nevertheless, the trials showed a reduction of recurrent polyps on aspirin compared with placebo. The COX-2 trials were stopped when their vascular adverse effects were made public.

This was encouraging randomised evidence, but although aspirin appeared to prevent polyps, only a minority of polyps become cancerous, and aspirin may be preventing the more benign ones, or the effect might not be maintained long term. Doubt about any effect of aspirin on risk of colorectal cancer was fuelled by the Physicians Health Study and the Women’s Health Study in the Unites States, two very large randomised studies of alternate day aspirin versus placebo in primary prevention of vascular disease that had not found any effect of aspirin on the ten-year risk of colorectal cancer. The only glint of possibility was that an effect might be delayed for longer than ten years because it takes about ten years to go from the early cellular and genetic changes in the colonic mucosa to the stage of presentation with a colorectal cancer and so it was possible that not effect would be seen until about ten years after aspirin was commenced.

**Figure 3.**
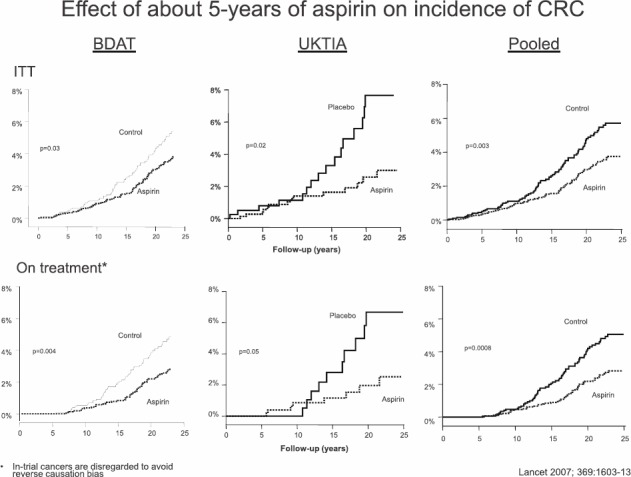
Effect of about five years of aspirin on incidence of colorectal cancer.

Prof Rothwell was collating the long term post-trial follow-up of the UK-TIA trial of about five years of aspirin versus placebo in the prevention of stroke and decided to use the opportunity to test the hypothesis that there might be a delayed effect of aspirin on risk of colorectal cancer by linking the trial data to cancer registration and death certification to capture post-trial cancers out to 20-year post-randomisation. He also persuaded Sir Richard Doll and Sir Richard Peto to allow him to do the same with the British Doctors Aspirin Trial cohort, a trial of about five years of aspirin versus control in primary prevention of vascular disease. In 2007, they reported the 20-year follow-up of these two trials and showed that in both cases the aspirin group had a reduced incidence of colorectal cancer from 10–15 years after randomisation (Slide). This was the first randomised evidence that aspirin does indeed prevent colorectal cancer. However, the doses used in these trials (around 300–1,200 mg daily) were higher than the low dose used to current practice (Figure 3).

### (BDAT, UKTIA pooled)

Rothwell therefore approached Tom Meade in London and Bo Norrving from Sweden to do the same long-term post-trial follow-up of their previous trials of low-dose aspirin versus placebo. In Tom Meade’s trial of aspirin, 75-mg daily was compared with placebo in primary prevention of vascular events in men with increased vascular risk (Thrombosis Prevention Trial—TPT) and the Scandinavian trial was in secondary prevention of stroke with a similar dose of aspirin. Again, 20-year follow-up was obtained via cancer registration and death certification and revealed a reduced long-term incidence of colorectal cancer in the aspirin groups.

Tom Meade had used a slow release aspirin in TPT that was thought to be almost fully deacetylated on the first pass through the liver, so that any action of aspirin was thought to be via effects on the platelets in the portal venous system and not in peripheral tissues or directly on the tumour itself.

With the bigger numbers available from four trials Rothwell and colleagues, they were able to look at the site of cancer within the colon and rectum, particularly proximal versus distal colon cancer. The greatest effect of aspirin was on risk of proximal cancer, with a much smaller or absent effect on distal colon and rectum. Given that rectal and distal colon cancers can be prevented by sigmoidoscopic and colonoscopic screening, which is much less effective in preventing proximal colon cancers, this observation was particularly important.

More recently, there has been Sir John Burn’s trial of aspirin versus placebo in Lynch syndrome, which has found the same extension of effect on colon cancer. Based on the extension of follow-up of the Women’s Health Study to 18 years, which showed no effect of aspirin up to ten years, Nancy Cook *et al* [3] have also now reported a similar delayed protective effect in the longer term risk of colon cancer, again with any significant effect confined to cancers of the proximal colon.

The Women’s Health Study also found trends towards reductions in oesophageal cancer and other GI cancers, similar to those seen in the longer term in the UK trials. Rothwell and colleagues had found substantial delayed reductions in long-term risk of oesophageal, stomach, and other GI cancers, as well as colon cancer.

One proposed explanation of the effect of aspirin that Prof Rothwell rejects is that the anticancer effect may be caused by its effect on bleeding—causing the cancers to be picked up earlier in a precancerous phase. However, there was no evidence of any earlier diagnosis of cancer in the aspirin groups in the trials studied and there was no reduction in the risk of GI cancer in the warfarin arm of the TPT despite the fact that warfarin caused more GI bleeding than aspirin.

Prof Rothwell concluded with a summary of the effects of daily aspirin:

Summary of effects of daily aspirin

(a) Non-vascular deaths reduced by 10–15%.
(b) Cancer deaths reduced by 20% > five years.
(c) Distant metastasis reduced by 30–50% > three years.
(d) Cancer incidence:

1. Possible overall reduction after five years? due to reduced growth/metastasis of existing cancers.
2. Reductions in long-term risk of colon cancer, oesophageal cancer, and other GI cancers > 5–10 years.
3. Limited evidence of effects on long-term incidence of non-GI tract cancers.

## The current situation worldwide: Bayer update on investigator sponsored studies

### Dr Elmar Detering, Bayer Pharma

Historically, aspirin has been developed mainly by independent academia and studies. Bayer’s colleagues are witnesses in the development of aspirin in various indications and new medical areas, and thus are deeply impressed and recognise the great significance of the news that continues to come out about this drug on a monthly or even weekly basis from investigators after more than a hundred years of its use.

Besides several clinical study types which are being conducted today in drug development, there is one study type of main interest for Bayer in the context of aspirin—investigator sponsored studies (ISS). The cardiovascular indications were achieved through ISS: in the past, Bayer has not performed its ‘own study’ in cardiovascular disease, but rather supported several of the large ISS in that area. However, currently, Bayer is the main sponsor of the ARRIVE trial in patients at moderate risk for cardiovascular events. For this study, all patients are recruited. Cancer is the secondary endpoint of this study, which will end in 2016.

Bayer is also in the process of assessing the possibility of submitting low-dose aspirin for the primary prevention of colon cancer at the European Medicines Agency.

In order to support the advancement of the scientific understanding of aspirin and its unique benefits, Bayer mainly supports ISS with drug supply (e.g. for ASPREE) and in some cases provides financial support, for packaging, (as is the case, e.g. for ASCEND and for CAPP3). The requests that are received for support of intended trials give Bayer a unique insight into where the research community’s interests lie. What is needed for approval to support a study with a drug is a final study protocol. Bayer receives these from all over the world from clinics or via local investigators through a local Bayer affiliate. They are passed to the global medical affairs department.

The following Bayer functions are involved in the internal discussion and decision to support a study with drug supply: Biostatistics, and global clinical development, HEOR, Medical Affairs, pharmacovigilance, regulatory affairs, and clinical pharmacology; regional marketing and medical affairs as well as the ISS-responsible people.

In 2012, of 242 ISS proposals reviewed by Bayer Pharma, 18 were rejected and 136 were approved without conditions. For the first half of 2013, of the 141 proposals reviewed, the corresponding figures were 18 and 73.

In 2013, we have decided to support ten new aspirin ISS mainly with drug supply.

The final approval of ISS can take from a few weeks to a few months. Once an ISS is approved, a contractual agreement is needed, which adds significantly to the timeline. Over all, taking past experience into account, it takes on average six months before the drug is delivered and the trial starts.

Dr Detering showed an overview of the ISS proposals reviewed in 2012 and 2013, first by indication and then by geographical distribution. They include ten therapeutic areas and 17 countries (including only those countries with a minimum of five proposals).

In 2013, Bayer Pharma received more new requests for the support of independent clinical studies with drug supply than in previous years. The following studies are being supported, mainly with drug and placebo supply:

ASCEND: using once daily aspirin and/or omega-3 fatty acids v placebo in patients with diabetes without existing occlusive arterial disease. Endpoint is reduction of cardiovascular risk.

ASPREE is in the United States and Australia, studying low-dose aspirin in the elderly with primary endpoints of death from any cause, incident dementia, or physical disability.

Biobank—biomarkers of cardiovascular disease.

ENVIS-ion—neurovascular imaging.

ALSOP—physical factors influencing quality of life such as eyesight, hearing loss, and sleep patterns.

SNORE-ASA—neurocognitive, radiological, and retinal effects of aspirin in sleep apnoea.

ASPREE—AMD to analyse and compare retinal images to determine if low-dose aspirin can prevent the onset or progression of macular degeneration (AMD).

ASPREE—to see if aspirin might reduce knee cartilage loss in older people.

SeAFOod (Systematic Evaluation of Aspirin and Fish Oil) studies polyp prevention in high-risk adenoma patients. The endpoint is colonoscopic recurrence of polyps one year after the initial colonoscopy.

ASCOLT is a multicentre randomised placebo controlled trial of aspirin for Dukes C and high-risk Dukes B colorectal cancers. The endpoint is the numbers of five-year survivors.

CAPP3, to start in 2014, is a randomised double-blind trial of aspirin in Lynch syndrome, looking at the cumulative incidence of primary colorectal cancers three dosage groups over five years. It will also look for endometrial cancers.

ACACC01 will be run by Prof Robert Benamouzig and aims to recruit 1100 patients into an open randomised trial of aspirin and curcumin in the prevention of colorectal cancer. It is planned to start in 2014.

AIDA (Adjuvant Intervention with Daily Aspirin) is scheduled to start in 2014: it is being run by Andrea DeCensi and his colleagues from Genoa and will have multiple centres throughout Italy.

LUNA, also by Prof DeCensi, will study aspirin and placebo in former smokers aged 50–75 years with an intermediate or high-risk miRNA signature and negative LDCT. Endpoints are the five-year total cancer incidence and mortality, major cardiovascular events, and total mortality.

Current smokers are the subjects in the NIH1 trial, at the University of Arizona, of lung cancer prevention. Endpoints include changes in gene expression in the nasal epithelium and in urinary PGE-M.

NIH2 is a randomised Phase II trial of low dose aspirin versus placebo in high-risk individuals with CT screen detected subsolid lung nodules called ground glass opacities (GGO). Dr DeCensi will describe it in detail.

ADD—Aspirin will add aspirin after standard primary therapy in early stage common solid non-metastatic tumours, including breast, colorectal, gastro-oesophageal, and prostate. Outcome is disease-free survival for the first two tumours, overall survival for gastrooesophageal tumours, and biochemical recurrence-free survival for prostate.

PROVENT will examine aspirin and/or vitamin D in preventing disease progression in men on active surveillance for low-risk prostate cancer. Follow-up will be radiological, biochemical, and histological.

AIDS trial: A pilot study has shown that aspirin has an effect on CD14 cells causing a delay in the shift towards the development of AIDS symptoms. This has led to a multicentre trial by the AIDS Clinical Trials Group (ACTG), the primary aim being the effect of adding aspirin to the patients’ standard ART therapy. The primary endpoint is the effect on plasma sCD14 levels, with secondary endpoints of any effect on various markers of immune activation, inflammation, and endothelial dysfunction.

COX-1 may be important in modulating neuroimmune systems. Dr Detering ended by quoting from Gu, Long *et al* (*Mol Brain* 2010, 11: 12):

‘There are now data indicating that expression of COX-1 is enhanced in neuroinflammatory disorders, including models of Parkinson’s disease and that COX-1 inhibition improves survival. The working spectrum of aspirin (targeting preferentially COX-1 rather than COX-2) may be the reason why it is an asset in the treatment of neuropsychiatric conditions associated with neuroinflammation and neuroprogression’.

This may lead to very interesting next steps for aspirin.

## Platelets, vascular disease, and aspirin in HIV

### Andrew Freedman, Reader in Infectious Diseases and Honorary Consultant Physician, Cardiff University

Dr Freedman’s talk covered the current natural history and prognosis of HIV infection, the causes of excess morbidity and mortality in the HAART era, ageing and comorbidities, vascular disease in HIV, and the role of platelets and of aspirin. It also covered the use of aspirin as a treatment for HIV.

HIV patients, he said, usually have a mild thrombocytopenia, and years ago, when Dr Freedman and his colleagues were working in Boston, they were interested in finding out whether HIV could infect megakaryocytes, and therefore that this might be the cause of the thrombocytopenia. It does not do so. In fact, it is usually an immune-mediated thrombocytopenia.

Thirty years ago when HIV was first identified, it was universally lethal, but we now have very effective treatment for it. We now have an ageing HIV cohort, so that other comorbidities, particularly vascular disease, are major causes of morbidity and mortality in HIV infected patients. Not much is known about the causes of vascular disease in HIV patients: there have been limited studies, including the role of platelets. The role of aspirin is still to be developed. Apart from its action on platelets, it may have a direct antiviral effect, and the talk ends with some evidence of this.

**Figure 4.**
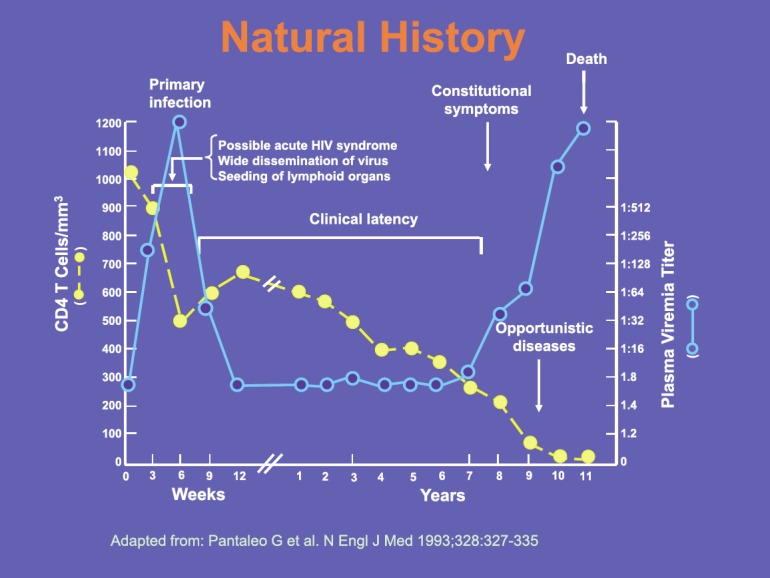
Natural history of HIV infection.

The slide shows the natural history of HIV in a patient in the absence of any treatment, which by and large no longer happens, though many patients are still diagnosed late in the course of the infection. Without treatment the interval between primary infection and death is typically around ten years, during which time there is a progressive decline in CD4 T cell numbers. When these reach around 200 cells/mm^3^, patients become susceptible to opportunistic infections such as pneumocystis pneumonia. We still see patients presenting with such infections, but ideally patients should be diagnosed much earlier in the course of their infection. Treatment can largely reverse the immune deficit, but is most effective if started before the onset of AIDS (Figure 4).

**Figure 5.**
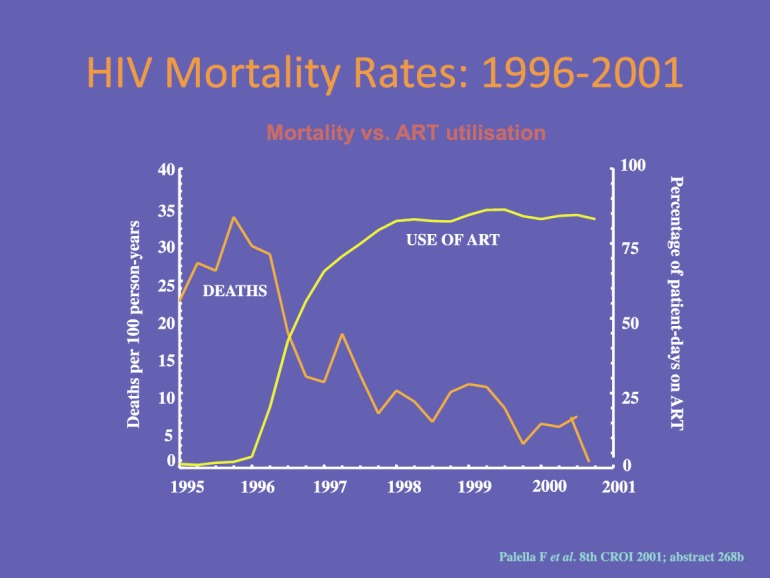
HIV mortality rate 1996–2001.

Although the first retroviral drug, AZT (zidovudine) was licensed in 1987, it was not until the advent of combination therapy (HAART) in 1996 that we achieved real success. The use of triple combination therapy, as opposed to mono or dual therapy, reduces the risk of drug resistance developing. Its introduction led to a massive reduction in HIV deaths from 1997 onwards so that there is now a very low mortality from HIV infection that has remained stable for more than ten years (Figure 5).

The UK CHIC cohort study [1] was one of several studies looking at life expectancy in patients with HIV. It published data on 17,661 UK adult HIV patients starting triple (ART) therapy with CD4 counts of 350 cells/mm^3^ or more. Seven per cent (1,248) died. The life expectancy increased by more than 15 years from 1996–1999 to 2006–2008, but was still 13 years less than in the general population. Starting ART late in the disease produces a 15-year reduction in life expectancy: starting ART when the CD4 count is <100 gives a life expectancy of 38 years; starting it when the CD4 count is 200–350 increased life expectancy to 53 years.

HIV prognosis in the HAART era is therefore now much better than in the earlier years. It is now a chronic treatable condition, but needs life-long prescriptions of multiple antiretroviral drugs. There is no cure as yet, but work towards a cure is ongoing. There is some evidence that HIV positive patients become frailer than the rest of the population as they grow older. Despite the success of treatment, their life expectancy is lower than that of HIV negative persons, to which malignancy, cardiovascular disease, renal disease, and liver disease all contribute.

### Cardiovascular disease in HIV

Many studies have shown that patients with HIV have an excess risk of MI. A Massachusetts study [2] comparing MI rates in patients with (3,851) and without (1,044,589) HIV infection showed an excess risk in HIV positive patients in all age groups.

There is a similar increase in risk for stroke. Chow *et al* of Boston [3] studied more than 4,000 HIV positive patients and matched HIV negative controls from 1996 to 2009. Among the HIV positive patients, the incidence of ischaemic stroke was 5.27 per 1,000 patient years compared with 3.75 in the HIV negative controls, a relative risk of 1.4. The relative risk was higher in younger patients and women. HIV was an independent risk factor after controlling for demographics and other predictors such as smoking.

Venous thromboembolic disease is also reported to be increased (by 2–10-fold in various studies) in HIV positive patients. The evidence is not as solid as for arterial disease in the literature, but Dr Freedman has seen in his practice many cases of thromboembolism, and histories of thromboembolic episodes in patients in the years before their HIV was diagnosed. Undoubtedly, the risk is higher in advanced disease, and the use of protease inhibitor therapy may be associated with increased thromboembolism. No one knows the mechanism of this connection: protein S deficiency, lupus anticoagulant, platelet activation, and endothelial injury are among those suggested.

There have been anecdotal reports of aspirin, as opposed to warfarin, being used in thromboembolic events, and being effective in preventing recurrence.

Why should there be an excess of vascular disease in HIV? Among the direct effects of the virus itself are dyslipidaemia, endothelial dysfunction, increased coagulation activity, and platelet activation. Even with good viral control, there still exists a chronic inflammatory state. The C-reactive protein (CRP) in HIV is usually elevated, mildly when it is well controlled, but it certainly does not return to normal levels. There may be many reasons for that. One contributor to it is translocation of bacteria across the gut wall.

Antiretroviral treatment may also contribute to vascular disease in HIV positive patients. Several anti-retroviral drugs cause hyperlipidaemia, and it is standard practice to monitor lipid levels in all patients taking them. There are probably other effects that are yet not understood.

Of course, ‘traditional’ risk factors are also present in HIV positive patients, particularly smoking. The majority of patients attending UK HIV clinics are smokers, and HIV physicians are probably not very good at addressing this. Diabetes is also more common among HIV positive patients, and this is an added risk for vascular events.

As for platelets, factors involved in platelet activation, such as soluble P-selectin and CD40 ligand, are elevated in HIV [4]. Authors have reported altered platelet morphology with giant platelets and vacuoles, which are indicators of platelet activation. One study has shown that although platelets are not infected by the virus, they can internalise it through the fibronectin receptor. Quite what effect that has on platelet function we do not know. What is known is that during antiretroviral therapy, the markers of in vivo platelet activation fall.

Treatment of HIV probably does reduce platelet activation, which in turn may reduce vascular risk. The virus itself has multiple effects on endothelium as well as on platelets, so that the overall effect on vascular risk is not simple to assess.

### Studies of aspirin in HIV

Meagan O’Brien and colleagues of New York studied the short-term effects of aspirin in HIV. They enrolled 25 HIV positive patients, all controlled on combination antiretroviral therapy, and 44 HIV negative controls. They were given aspirin 81 mg daily for one week, and their platelet function before and after the week was assessed for spontaneous aggregation and aggregation induced by adenosine phosphate and arachidonic acid (AA). They measured markers of T cell (CD38 and HLA-DR) and monocyte (sCD14) activation. Other tests included CRP, IL-6, and D-dimer.

At baseline, platelet activation was significantly increased in the HIV positive group over that in the controls. Platelet aggregation fell in all subjects after one week of aspirin, in response to ADP and AA, but the response to AA, but not to ADP, remained significantly higher in the HIV patients than in the controls. There is increasing interest in the chronic inflammatory state as a cause of the various long-term health complications such as vascular disease and bone mineral density in HIV patients. Disappointingly, in view of this, although there was a significant fall in cellular activation markers in the HIV patients, there was no fall in CRP, IL-6, and D-dimer.

The conclusion from this pilot study was that the heightened platelet and immune activation in treated HIV patients is attenuated by aspirin. The benefits of aspirin should be studied further in HIV both for its anti-thrombotic and immune-modulatory effects, and a larger trial, run by the ACTG, is being planned.

The prescription of aspirin in HIV patients is not yet ‘on the radar’ for many HIV specialists, including Dr Freedman himself. They are quick to prescribe statins for hyperlipidaemia, but none of the USA, UK, or European guidelines on HIV treatment include aspirin. A recent study in Birmingham Alabama of nearly 2,000 patients attending an HIV clinic found that 400 of them qualified to receive aspirin by the U.S. Prevention Task Force Criteria guidelines [6]. They were based on the ten-year Framingham CHD risk score for men, and the ten-year Framingham stroke risk score for women, above 10%.

Of the 400, with a mean age of 52, and of whom 94% were male, only 66 (17%) were being prescribed aspirin. It was concluded from this and from a more recent study presented earlier this year that aspirin is markedly underprescribed.

Finally, there is fairly weak evidence that aspirin may have a useful direct effect on the HIV virus. One study not using aspirin, but the salicylate O-(acetoxyphenyl)hept-2-ynyl sulphide dose dependently inhibits HIV replication in lymphocytes and macrophages *in vitro* [7]. It did not affect the entry of virus into the target cells or the later stages of viral replication, but it was suggested that aspirin might act as a reverse transcriptase inhibitor.

This is probably not highly relevant clinically now that there are effective anti-retroviral drugs for HIV, in five different classes (there are now around 25 drugs licensed for anti-HIV treatment). This was in 2003, but there was a news item in *Nature* in 1993 (remember that it was not until 1997 that there was effective combination therapy against HIV) about a proposed study in New York planning to give 4 g/day of aspirin to 46 asymptomatic HIV-infected patients. They were convinced at the time that it would be useful for these patients. Sadly, nothing more is known about this study—it did not make it to publication.

## 

References1.MayMImpact of late diagnosis and treatment on life expectancy in people with HIV-1: UK Collaborative HIV Cohort (UK CHIC) StudyBMJ2011343d601610.1136/bmj.d601621990260PMC31912022.TriantVAIncreased acute myocardial infarction rates and cardiovascular risk factors among patients with human immunodeficiency virus diseaseJ Clin Endocrinol Metab200792725061210.1210/jc.2006-219017456578PMC27633853.ChowFCComparison of ischemic stroke incidence in HIV-infected and non-HIV-infected patients in a US health care systemJ Acquir Immune Defic Syndr20126043511810.1097/QAI.0b013e31825c7f2422580566PMC36700864.GreselePEndothelial and platelet function alterations in HIV-infected patientsThromb Res20121293301810.1016/j.thromres.2011.11.022221921575.O’BrienMAspirin attenuates platelet activation and immune activation in HIV-1-infected subjects on antiretroviral therapy: a pilot studyJ Acquir Immune Defic Syndr2013633280810.1097/QAI.0b013e31828a292c23406976PMC37564896.BurkholderGAUnderutilization of aspirin for primary prevention of cardiovascular disease among HIV-infected patientsClin Infect Dis201255111550710.1093/cid/cis75222942209PMC34918607.PereiraCFAspirin-like molecules that inhibit human immunodeficiency virus 1 replicationAntiviral Res200358325363310.1016/S0166-3542(03)00006-8127674738.MacilwainCAspirin on trial as HIV treatmentNature1993364643636910.1038/364369b08332198

## Add-aspirin and other treatment trials

### Ruth Langley, Medical Oncologist, UK Medical Research Council Clinical Trials Unit, University College London

Dr Langley outlined the structure of her talk. She would focus on aspirin for the treatment of cancer, rather than prevention. She would outline the framework and the normal pathway for the development of anticancer drugs, which may explain why aspirin may have been overlooked as an anti-cancer agent. She will summarise the evidence for using aspirin as anti-cancer therapy, highlighting data on cancers other than colorectal cancer. She will then cover the add-aspirin trial and the challenges it posed for her, and conclude by discussing the potential mechanisms of aspirin’s anticancer action.

In drug development trials, particularly in oncology, we normally start with promising preclinical data, then move to the Phase 1 study, the first use in humans. In Phase II oncology trials, we are looking for efficacy and the dose we would like to use. The studies are often in advanced disease: they are small, non-randomised, looking for response rates judged usually by tumour shrinkage. If there is a response, we then move on to Phase III studies in which there is a comparison with standard therapy. Initial Phase III studies are often in patients with metastatic disease.

There is a plenty of preclinical data for aspirin *in vitro* and *in vivo* supporting its role as an anti-cancer agent. We know we can use it in humans because we have been doing so for many years.

However, if we give aspirin to patients with established metastatic disease for four weeks, we are very unlikely to see any tumour shrinkage. Even if we compare it with standard therapy and start with metastatic disease, the effect of aspirin (as we have heard today) will take many years, and the patients with widespread metastatic disease may die before that time. This is probably why aspirin has been about for many years but has not before been used by oncologists.

We are now beginning to realise that we have to rethink the evaluation of some new therapies, particularly those that may be effective when the tumour burden is minimal and therefore difficult to evaluate. The issues are not unique to aspirin: they are potentially similar for COX-2 inhibitors, statins, and metformin among other common drugs that have been around for years that might have some anticancer activities.

There is plenty of evidence for using aspirin as an anticancer therapy: they include

(a) the *in vivo* data of Gasic *et al* and many *in vitro* mechanistic studies;
(b) epidemiological data of primary prevention across tumour sites summarised by Bosetti *et al*;
(c) randomised data showing primary prevention—CAPP2 trial, cardiovascular studies and the Women’s Health Study;
(d) randomised data from prevention of colorectal adenomas;
(e) non-randomised data in the adjuvant setting across tumour types.

The Gasic studies injected fibrosarcoma cells into the tail vein or the hind leg of mice and they gave half the group aspirin in their drinking water and half regular water. There was a significant reduction in lung metastases in the aspirin group. If we had this kind of data from new agents coming through today, everyone would be very excited about it. This study was published in 1972 in *The Lancet* [1972, 2(7783): 9323–3]. Most of the oncologists have never seen these data.

Discussions on the epidemiological data for aspirin in primary prevention of cancer usually focus on colorectal cancer. From a comprehensive 2012 review, Dr Langley selected data on other cancers. For example, aspirin was associated with an almost 30% reduction in risk of gastric cancer and the risk reduction was similar for oesophageal cancer. There is also potentially important reduction in risk for breast, lung, and prostate cancers.

Newer data (most of the studies have been published in the last two years) are coming from aspirin given after cancer is diagnosed in non-randomised studies, but in large epidemiological cohorts. Three large colorectal studies have all shown similar results—reduction on colorectal cancer mortality and improved survival. There have been similar results in breast cancer, and in prostate cancer, people are starting to interrogate their databases. It seems that aspirin given after prostate cancer diagnosis may also be effective.

There has been one interesting Chinese study in gastro-oesophageal cancer. It is described as a randomised study, but after reading it, it does not fulfil the usual definition of randomisation. After oesophagectomy one of three things happened. Patients were sent to one of three post-operative wards. It was not clear how people were allocated to each ward, but in ward 1, there was no further anticancer treatment. In ward 2, patients were given aspirin, and in ward 3, they were given placebo.

The very preliminary results from this trial suggested that aspirin given after oesophagectomy improved five-year survival.

Peter Rothwell’s data show that from the randomised cardiovascular trials, if you were given aspirin, this decreased the risk of developing metastasis that had not been presented diagnosis. The data are from five randomised studies with about 17,000 participants. The effects were seen across tumour types, particularly adenocarcinomas, and across sites of metastases such as lung, liver, and brain.

All this evidence persuaded Dr Langley’s group to design four parallel Phase III studies with one overarching protocol. All the patients have to have undergone primary treatment with curative intent for an early stage common solid tumour. There is an eight-week run in period of taking aspirin 100 mg daily. There are four individually powered studies—colorectal, breast, gastro-oesophageal, and prostate—and for each cohort, subjects will be randomised to placebo, aspirin 100 mg or aspirin 300 mg daily for at least five years. Each cohort has a primary outcome measure appropriate to the disease site. This is disease free survival in the colorectal and breast cohorts, overall survival in the gastro-oesophageal study (these are patients likely to have metastases early on after surgery), and biochemical failure in the prostate cancer patients.

The patients will be followed particularly closely for the first five years, after which there will be long term follow-up because many of the effects of aspirin do not appear for many years after diagnosis.

Where is the project today? There is now funding from Cancer Research UK and from the Department of Health (subject to contract). It will take many years. Bayer has agreed to provide the aspirin and placebo, and the first patients should be enrolled in 2014. It is planned to do the trials primarily in the UK, but for the gastro-oesophageal and the breast cohorts patients will be enrolled in India as well.

The rationale for the drug is that:

(a) it is a low-cost generic drug available worldwide;
(b) accessible in low resource settings (unlike many new agents or complex regimens) where incidence of cancer is increasing;
(c) it has low toxicity and a known safety profile;
(d) there is a possible therapeutic role for several of the most common cancers;
(e) it has potential for huge global impact on cancer outcomes.

Discussions are ongoing about the possibility of adding aspirin as an adjuvant agent, using a factorial randomisation, once other arms are confirmed in other disease sites. It is increasingly being recognised that if the benefits being seen with aspirin in the epidemiological studies are correct, aspirin must be evaluated in clinical trials. Patients in clinical trials will be taking aspirin for other reasons, and therefore is a potential confounding factor.

There are challenges for aspirin as an anticancer treatment. There is still a huge resistance to using aspirin even if we hypothesise that there are large potential benefits, because of the concerns about bleeding. We will have to work very hard to give a clear, fair, and balanced message about its toxicity. It is hard to estimate how many bleeds are likely to occur in Dr Langley’s proposed trial. A meta-analysis from the Anti-Thrombotic Trialists Collaboration involving six primary prevention studies with around 95,000 participants, mean age 56 years, 46% of whom were men, showed that the risk of a G-I bleed or a bleed from another extra-cranial site was about seven per 1,000 per year in the control group, going up to ten per 1,000 per year on aspirin. If this is related to what is expected during the study, it is expected that there will be 11 bleeds in the placebo arm and 35 in the aspirin arm. This is with a 2:1 aspirin:placebo randomisation.

Explaining these figures to potential investigators has been helpful.

Other issues include adherence. It is difficult to ask patients to take a tablet for five years, particularly in the trial setting. One would think that patients who have had a cancer are particularly well motivated to take a medicine that in theory will prevent it from returning. Yet, data with drugs such as tamoxifen in breast cancer show adherence is not always good.

There has been a suggestion that aspirin should be taken for cancer prevention by everyone in a certain age group. If that does happen, that could be an issue for the trial. Another issue is the perspective on trials in oncology: it is unusual to test an agent in the adjuvant setting without proven benefit in metastatic disease.

There should also be caution about interpreting data on single mutations and aspirin efficacy particularly if based on small numbers.

### Possible anti-cancer mechanisms of action of aspirin

Dr Langley ended with a review of the possible mechanisms of anti-cancer action of aspirin. The anti-metastatic effect is probably through platelets. There has been much debate on whether or not a single daily dose of aspirin can have a direct effect on systemic tissue. Carlo Patrono has suggested that aspirin first affects the platelets and that subsequent interaction between the platelets and COX-2 in the tissues might explain why it appears that both aspirin and COX-2 inhibitors are good anticancer agents. NFkB may be involved as may the anti-inflammatory reaction of the whole body to tumours. There may be some effect of natural salicylates, but the fact is that we do not yet know the details of aspirin’s effects on tumours.

## Aspirin and colorectal screening

### Prof Richard Logan, Director of the Eastern Bowel Cancer Screening Hub, England; Division of Epidemiology and Public Health, University of Nottingham

Dr Logan posed the question ‘What place is there for aspirin chemoprevention in the bowel cancer screening era?’

Worldwide colorectal cancer is the third most common cause of cancer mortality, after lung and breast cancer. Half of all European countries have either started bowel cancer screening programmes or are in the process of doing so. Most of the wealthier European countries have started screening, and in the United States, Canada, and Australia, screening has been going on for over ten years.

In the UK incidence of bowel cancer, incidence has risen slightly in men, which may well reflect improvements in cancer registration, but remained steady in women up to 2003. In the last five years, there has also been a slight increase in incidence in women, which may be due to withdrawal of HRT and/or due to screening, which started in 2007–2008. Nevertheless, despite these small increases in incidence there has been a steady decrease in bowel cancer mortality in both men and women.

At the time, Dr Logan qualified as a doctor in the 1970s, bowel cancer had a dismal prognosis in the UK with fewer than 30% of patients surviving five years from diagnosis. Since then there has been a steady increase in survival with the latest figures, from 2005 to 2009, show survivals in the mid-50s per cent. While there has been much criticism of cancer survival in the UK (Sir Mike Richards has used these figures to fight for improved services), we are now approaching the best of what other European countries achieve. Paradoxically these improvements reduce the need to consider preventive measures

The traditional public health approach to bowel cancer prevention can be summarised as advising people not to smoke, drink, eat red meat, be fat, or be slothful. Thus, in the absence of being able to convince people to follow this advice, chemoprevention has an obvious attraction.

Nevertheless, as with any preventive approach in medicine, events prevented are not readily identifiable and adverse events. Colorectal cancers prevented are invisible while it is all too easy for the physician involved to say ‘that patient has had a GI bleed’ a condition with a 5–10% mortality rate. Bear in mind, too, that bleeding from the upper GI bleeding becomes much commoner as people age and that it is now clear that the comorbidity for which many people are prescribed low-dose aspirin is in fact itself a risk factor for developing upper GI bleeding.

Thus, there is considerable reluctance among gastroenterologists to consider aspirin chemoprevention in the average risk population when there is such widespread acceptance and support for screening being effective whether by endoscopic methods or stool testing.

So, how effective might screening be for bowel cancer? A paper in the 19th September issue of the *New England Journal of Medicine* (*NEJM*) reported on the 30-year follow-up of the Minnesota faecal occult blood screening trial (Shaukat *et al* [2]). The outcomes measured were deaths from any cause and from colorectal cancer in three groups each of 15,000 people. The trial reported a 32% reduction in risk of mortality from colorectal cancer on annual screening, and a 22% reduction on biennial screening compared with the reference number on no screening. The comparative figures for colorectal cancer deaths in the three groups were 200, 237, and 295. Given these small differences in absolute numbers of deaths, it is not surprising that bowel cancer screening had no detectable effect on all-cause mortality. There was a hint, even, that the all-cause mortality was a little higher among the people undergoing annual screening. This observation has been a cause of debate among gastroenterologists in the past.

One feature of the Minnesota trial is that the test used for faecal occult blood involved rehydrating the kit before testing for blood, instead of testing the dry specimen. This increases the sensitivity but reduces the specificity, so that colonoscopies were done in 38% of the population subjected to annual screening and to an extent makes this arm a trial of limited colonoscopy screening. Even in the biennial arm, the colonoscopy rate arm the rate at around 16% was much higher than in the European trials. These figures are not generalisable to what is happening in the UK, where the colonoscopy rate after screening is much lower at 2%.

Dr Logan presented estimates of mortality reductions by screening using the current UK model of two-yearly screening for guaiac faecal occult blood testing and compared them with systems used in other countries. In the UK, in which the estimates are based on the people invited to attend, the current reduction is 15–16%. This rises to around 25% when based on a per protocol analysis (of the people who take up the screening), so even then the reduction in colorectal cancer mortality obtained is still not enormous.

The UK programmes are likely to introduce a faecal immunochemical test that is much more sensitive and easier to do. The evidence at present, from modelling alone is that it should produce a 36% reduction in bowel cancer mortality.

The Flexi-sig trial was reported by Wendy Atkin in April 2010. It showed a 31% reduction in bowel cancer mortality among the invitees, rising to 43% for subjects who underwent the flexible sigmoidoscopy. These results were so striking that, within six months, the UK Prime Minister David Cameron had announced to the country that flexi-sig screening programme was to be introduced throughout England by 2015 the time of the next election. Six pilot sites have now been in operation since the summer of 2013.

Three other trials of flexible sigmoidoscopy screening have now been reported. All found significant reductions in colorectal cancer mortality with the Italian trial reporting reductions of 22% overall and 38% per person and the US PLCO trial a reduction overall of 26%. These two trials like the UK trial screened people who had already expressed an interest in screening, which may inflate the effectiveness of screening as screening uptake tends to be higher than in trials of the general population. The Norwegian trial in which the general population was invited to be screened gave a reduction of 17%. From these results, it is clear that Flexible sigmoidoscopy screening is at least as effective as the current guaiac faecal occult blood screening in reducing mortality.

Colonoscopic screening is much more prevalent in North America but it has been introduced without any randomised trials of its effectiveness. Several trials are now in progress but until these report in over ten years time the only evidence of its effectiveness comes from observational studies. Case-control studies have estimated a 43% reduction in bowel cancer mortality. Modelling studies have suggested that a colonoscopy every ten years will reduce mortality by 64%, so that regular colonoscopy for the population is the preferred screening in the United States.

In the same September issue of the *NEJM*, there was an analysis from a combination of the Nurses’ Health Study (all women) and the Health Professionals Study (mostly men), which gave the hazard ratios for various procedures including that for polypectomy. Among people reporting a polypectomy, there was a 40%: for a negative sigmoidoscopy, it was 41%, and negative colonoscopy 56% reduction. So, these findings were in keeping with earlier estimates.

These figures were impressive, but tumour site matters. There is evidence that colonoscopy is not so successful for right-sided lesions. Flexible sigmoidoscopy has, as would be expected, no protective effect against lesions in the right side of the colon but is impressive for the left side of the colon. Colonoscopy, too, is less effective in protecting against right colonic lesions: it gives a 27–28% reduction in mortality from them against a 76% reduction in mortality from left-sided lesions. Nonetheless, it does not leave much scope for chemoprevention if the choice for screening is colonoscopy.

As for polypectomy (which gastroenterologists seem to enjoy doing!), the data that regular colonoscopic surveillance and polypectomy results in a substantial reduction in bowel cancer incidence rather than mortality are not impressive. In the same Minnesota trial among people having regular colonoscopy with polypectomy, there was a 17% reduction in incidence of bowel cancer after 18 years.

In the Nottingham trial, using unhydrated guaiac faecal occult blood tests to determine the need for colonoscopy, only 2–3% of those screened needed colonoscopy and their reduction in bowel cancer incidence was only 3% after 20 years. So, in the UK, we are spending a lot of time and resources removing polyps but current evidence suggests that doing this within the UK screening programmes will have only a modest impact on bowel cancer incidence.

So, in comparison, how effective might bowel cancer chemoprevention be? Aspirin, at present, is the only agent for which there is sufficient evidence to consider using it for chemoprevention in people at average risk of bowel cancer. While the COX-2 inhibitors do have a chemopreventive effect, their cardiovascular side effects render them unacceptable for use in the average risk population. Calcium also has a chemopreventive effect, but there is concern over a possible increase in risk of MI. As for selenium, vitamin D plus calcium and curcumin, their efficacy is not yet established. A trial of eicosapentanoic (fish oil) is being done within the screening programme in England.

The effect of aspirin on the long-term risk of cancer of the proximal colon is much more striking than that on the distal colon as shown by the papers of Peter Rothwell and recently confirmed in the Women’s Health Initiative aspirin trial. The same applies to mortality: indeed its effect on mortality appears to be greater than its effect on incidence. If that is correct and colonoscopy is much less effective for the right colon, then aspirin chemoprevention is a very suitable course to take in people who are at increased risk of right colon disease. Supporting this, it is extremely encouraging now to have the data from the Women’s Health Trial confirming the lower cancer risk beginning ten years after the start of low-dose alternate-day aspirin treatment.

What about the combination of chemoprevention with screening? While this is an attractive approach at present, the only data on this come from modelling studies. The GUT paper published in 2011 took the very best of the evidence for aspirin efficacy and reasonable evidence for the effectiveness of colonoscopy: the model found that if people are given aspirin and also offered colonoscopy, there was an extra 13% reduction in mortality (from 68% to 81%).

A criticism that could be made of this study is that they only lagged the effect of aspirin by five years and it is now clear from Peter Rothwell’s study and from the Women’s Health Study that almost ten years are needed to pass before the effect of aspirin on colorectal cancer incidence is evident.

A combination with aspirin prevention looks more promising for flexible sigmoidoscopy. The model produced a 39% reduction in bowel cancer mortality on flexi-sig, and adding aspirin almost doubled that (to 69%). This is an area deserving further research. The Bowel-Scope programme as it is called in the UK is now under way and finding some way of studying aspirin within has obvious attractions.

In planning trials, the estimate used for bowel cancer reduction by aspirin alone is 41%, which Dr Logan considered high but the data from Peter Rothwell and the Women’s Health Study now make this figure appear reasonable.

This presentation has only considered the place of chemoprevention in people at average risk of colorectal cancer who currently are being offered screening. As for people who are at high (lifetime risk 80–100%) or moderate (5–15%) risk because of their family history, Dr Logan did not feel it reasonable to tell them to wait for twenty years until we have the answer. He tells them that if they wish to take something to prevent cancer then aspirin is worth taking.

In Prof Logan’s opinion, the outstanding questions on aspirin to prevent bowel cancer are:

(a) What dose is needed? While it is now clear that low-dose aspirin is effective, it is unclear whether this is the optimal dose or whether the balance of risk-benefit favours a 150- or 300-mg dose.
(b) For how long? Unfortunately, we have to tell people that they need to take aspirin for ten years before it works. This remains the major drawback to recommending aspirin chemoprevention to people at average bowel cancer risk.
(c) How effective is aspirin when combined with other drugs such as calcium and selenium? We need to know much more about them. If the current fish oil trial gives the result that the combination of fish oil and aspirin gives a reduction in mortality of 60%, then this would make chemoprevention a very attractive option but at present aspirin alone gives a reduction of around a third, and the addition of fish oil will have to offer significantly more than that to make people consider it.
(d) What is the balance of benefit and risk? This will follow in the next paper, given by Ceri Phillips.

## 

References1.NishiharaRWuKLockheadPLong-term colorectal-cancer incidence and mortality after lower endoscopyN Engl J Med201336912109510510.1056/NEJMoa130196924047059PMC38401602.ShaukatAMonginSJGeisserMSLong-term mortality after screening for colorectal cancerN Engl J Med20133691211061410.1056/NEJMoa1300720240470603.CookNRLeeIMZangSMAlternate-day, low-dose aspirin and cancer risk: long-term observational follow-up of a randomized trialAnn Intern Med20131592778510.7326/0003-4819-159-2-201307160-0000223856681PMC37135314.HassanCRexDKCooperGSPrimary prevention of colorectal cancer with low-dose aspirin in combination with endoscopy: a cost-effectiveness analysisGut20126181172910.1136/gutjnl-2011-300206219975455.HullMASandellACMontgomeryAAA randomized controlled trial of eicosapentaenoic acid and/or aspirin for colorectal adenoma prevention during colonoscopic surveillance in the NHS Bowel Cancer Screening Programme (The seAFOod Polyp Prevention Trial): study protocol for a randomized controlled trialTrials201314123710.1186/1745-6215-14-23723895505PMC37336946.ChanATAspirin in the chemoprevention of colorectal neoplasia: an overviewCancer Prev Res (Phila)2012521647810.1158/1940-6207.CAPR-11-039122084361PMC3273592

## Benefit–harm balance and cost effectiveness

### Prof Ceri Phillips, Head of Swansea Centre for Health Economics

The meeting has concentrated on the role of aspirin, the evidence for its efficacy, and where and how it works. Prof Phillips, as a Health Economist, wanted to restore a sense of balance, looking at ratios and costs. Healthcare systems have enhanced quality of life and increased life expectancy resulting in a greater proportion of people living to a grand old age and at the same time putting increasing demand on the limited resources that are available for healthcare systems.

People’s expectations about what health professionals can do to them and for them have also increased over time. The notion of the healthcare dilemma has sought to explain the exponential increase in demand for healthcare services against a background of constraints on available resources with some systems facing a decrease in resources in real terms.

We therefore have to manage the levels of demand for healthcare with the resources available. Resources are channelled into interventions some of which work, some do not work, and some we do not know whether they work or not. The term ‘efficiency’ is used to assess the extent to which limited resources can be allocated to maximise the benefits for society. In some cases, this means spending more money to get greater benefit. Being efficient is not, as politicians believe, just about making savings. It is about using the resources that are available in the most effective and best possible way for the patients.

A famous Health Economist, Alan Williams, of York, challenged an audience of healthcare professionals in 1993. He stated that they ‘have a duty to establish not only that they are doing good, but that they are doing more good than anything else that could be done with the same resources’. If we could look at our ‘end of term school report’, we could all admit that we could do better with the resources that are available. Williams also indicated that healthcare professionals not being efficient meant that resources are wasted: it could end with the wrong people being treated or people being treated inappropriately or even dying unnecessarily.

Since the late 1990s, health technology assessment has become an industry that has grown all over the world. It brings together the evidence of effectiveness, costs, and the broader impact of healthcare interventions on society. It is the industry which NICE forms part of, so when NICE is appraising a technology it is undertaking a health technology assessment. Health economics assessments are a critical component of the process of evaluation. Cost effectiveness seeks to evaluate the value of intervention programmes such as screening, to see if they constitute value for money. What health economists are keen to establish is not just whether an intervention works but to what extent does it represent an efficient use of resources—in other words, does it deliver value for money?

NICE requires evidence on effectiveness of interventions, their safety, balancing benefit against harm, but also evidence of cost effectiveness. In assessing the costs, those associated with dealing with the harm must be added to the costs of delivering the benefits. Value means are we prepared to incur the risk in order to gain the particular benefit.

The key question is how much more effective is the treatment being studied, in order to justify a greater cost. We are not looking at averages, or mean effects, or mean effects plus confidence intervals, but at incremental effects.

In relation to aspirin, in its role in primary prevention, it is evident that its efficacy is clear. It generates reduced risk for cancer and cardiovascular events. What is less evident is which groups of patients it benefits. The alternative to aspirin use is to do nothing, and raises the question of to what extent potential harm offsets potential benefits.

We need to establish this benefit–cost ratio. In any intervention, we must assess all of the costs associated with the implementation of a primary prevention strategy.

Furthermore, we need to be absolutely clear on what the benefits of the intervention actually are. We may well find that resources can be released that can be used for other patients. There will be benefits in terms of additional life years that the patient can enjoy, which can be measured as quality-adjusted life years, which NICE uses as a measure of effectiveness. However, we also have to be cognisant of the fact that there may be bleeds and that there may be issues relating to social and political acceptability of the intervention.

The overall impact of increasing aspirin use should be measured—including the reduction in cancer risk and in cardiovascular risk. The reduction in costs further downstream because of events prevented need to be determined. The U.S. Preventive Service Task Force in 2009 commented that ‘aspirin should be used for primary prevention in men when the potential effect of a reduction in myocardial infarction outweighs the potential harm of an increase in gastrointestinal haemorrhage’. Note the word ‘should’ rather than ‘could’.

A few years ago, the Swansea health economics group looked at the use of aspirin in a population with a series of risks for cardiovascular disease. They looked at the risk factors according to age, gender, and other factors such as smoking, diabetes, blood pressure, and cholesterol. They estimated the life years that would be gained with the increasing use of aspirin in people with varying degrees of risk, and offset these benefits against the potential harms that might emerge.

They assessed the cost per cardiovascular event avoided and the cost per life year gained if aspirin use could be raised from 55% to 75% of the eligible population. There would be cost savings in men and women aged 55 years and over, but for the younger population, the costs per event saved and per life year gained were generally reasonable except for the lowest age category (Table 2).

It was concluded that aspirin prophylaxis in the over 50s was indeed value for money.

The results seemed to confirm that the use of aspirin in preventing cardiovascular events is cost effective at five-year risk rates of 3% and would be cost saving at five-year risk rates of 5%. However, the results are very sensitive to the cost of the aspirin preventive strategy and the costs and risks of adverse events. This caveat caused some concern when it was published, and was a major issue that was a block for many physicians in making their decisions. At the time, Peter Elwood stressed that the protection was not only about cardiovascular events but about cancer, too.

In relation to the effect of aspirin on cancer risk, one study has become particularly helpful. Michael Pignone and colleagues’ paper in the *Journal of General Internal Medicine* in 2012 was titled ‘Effect of including cancer mortality on the cost-effectiveness of aspirin for primary prevention in men.’

It highlighted the objective of whether including a cancer mortality-reducing effect influences which men would benefit from aspirin for primary prevention. It involved the study of 10,000 45-year-old men with a 5% ten-year CHD risk. Prof Phillips highlighted the areas in which there are increased benefits and reduced costs. Aspirin led to 110 more G-I bleeds than no aspirin, which is to be expected. There were 51 fewer MIs in the aspirin arm than on no aspirin. Overall, in each case, a ten-year perspective was not long enough to assess the benefits of aspirin over the harm from adverse events.

However, taking a 20-year perspective and a lifetime perspective, the benefits were clearly evident. At 20 years, without including the protection against cancer, there is still a very small negative impact in terms of QALys. Nevertheless, there remains a degree of confusion.

This is where the confusion lies. Using cost-QALy estimates in 45- and 55-year-old males with different levels of risk highlights the issue. In the case of a 45-year old male with low cardiovascular risk and without adding in the cancer risk, the harm of taking aspirin would outweigh the risk and aspirin would not be used. When the risk of cancer mortality risk is added, the potential benefit now outweighs harm and aspirin would be given, resulting in a positive cost–benefit ratio, although whether we would wish to pay that is another issue.

In people at higher risk, aspirin is more effective and less costly, so they are in a win–win situation in terms of benefit relative to harm.

In the case of a 55-year-old man, there is relatively less benefit because there is less time for the benefit to occur, take place, but there is still a positive cost–benefit ratio because it represents value for money, compared with the US government threshold of US$50,000 per life year gained.

Probabilistic sensitivity analysis suggests that aspirin is cost saving or cost effective (in that it costs less than $50,000 to save one life year) in 59% of scenarios when only cardiovascular protection is estimated. When the same analysis is enlarged to include also cancer prevention, it is cost saving or cost effective in 96% of scenarios.

We have therefore shifted from an element of doubt to certainty that aspirin is cost effective when the anticancer effect is factored into prevention.

Conclusion: It is clear from this that the added potential beneficial effect of aspirin on cancer has increased the probability that it represents a cost-effective primary prevention strategy for CVD and cancer.

Prof Elwood added in the discussion afterwards that it is usual for people to ask their doctor’s advice on whether or not they should take aspirin, and most of the doctors do nothing about it. He suggested that doctors would reduce harm by first asking about stomach problems and measure blood pressure before prescribing aspirin and that this would substantially reduce the risk of harm. Prof Phillips agreed that it would but added that the need to investigate stomach or blood pressure complications would add to the cost and therefore may make the treatment less cost effective!

## Studies on lung and colon cancer in italy

### Dr Andrea DeCensi, Head, Division of Medical Oncology, Galliera Hospital, Genoa

Using drugs like aspirin is, for medical oncologists like myself or Ruth, a good way to unify the dissociated personality that a medical oncologist has, in the sense that when I am in Genoa I am Mr Hyde giving terrible poisons (chemotherapeutics) to sick people, whereas when I study aspirin (I have spent all my career studying tamoxifen and metformin and other non-cytotoxic agents to prevent or treat cancer) I am more like Dr Jekyll. When I and Ruth, I suppose, turn to aspirin we are facing a paradigm shift in medical oncology as we try to treat cancer with a more physiological approach.

I will briefly outline our randomised adjuvant trial of low-dose aspirin in colon cancer, then I will talk about two studies of low-dose aspirin in people at high risk of lung cancer who are undergoing a CT screening programme.

Low-dose aspirin has been shown to prevent the recurrence of adenoma, particularly those at high risk of progression to cancer, with the so-called advanced lesion with high grade dysplasia or tubular villous histology. Ten years ago, it was shown that the dose response relationship may not exist in colorectal carcinogenesis. We have already heard about the different mechanisms of action of aspirin on colorectal carcinogenesis such as the prevention of polyp formation or progression to dysplastic polyps. The paradigm shift has been the work of Rothwell and colleagues in the potential use of aspirin in the treatment setting, in particular, the prevention of metastasis.

The role of platelets in metastasis formation appears to be critical particularly in the first days or weeks of carcinogenesis progression. The role of platelets in aggregating the circulating cancer cells will mask the cytolytic effect of natural killer cells. This allows adhesion to neutrophils, which has important consequences for the invasion of the endothelial cells by the tumour cells.

There are preclinical models that show that this effect occurs a few hours after the presence of cancer cells in the bloodstream. A 2010 paper by Rothwell *et al* was shocking in that it showed that low-dose aspirin had a greater effect on mortality than on incidence of colorectal cancer although it was restricted to the proximal colon. This supported the contention that aspirin could prevent metastasis formation.

In a subsequent subgroup analysis of that meta-analysis, it was shown that aspirin could reduce the risk of later metastasis after the initial diagnosis of colon cancer by 75%. A dose of less than 300 mg exerted the same anti-metastatic effect and anti-mortality effect as a higher one.

With this background, Dr DeCensi’s team have designed the AIDA trial, which follows the Genoa tradition of naming their trials after famous operas. They have already conducted the three or six colon adjuvant (Tosca) trial that addressed the issue of the duration of chemotherapy after the diagnosis of stage 2/3 colon cancer. Over 3,500 patients were randomised to three or six months of chemotherapy, which is the current standard. The chemotherapy is based on two drugs, fluoropyrimidines and oxaliplatin, which is a very neurotoxic drug, so the issue of the duration can be really important.

In this context, we have a big group of over 100 centres in Italy who completed their recruitment very quickly and we are now proposing that they add aspirin to patients who are at risk for recurrence for which most of them will be given chemotherapy. This is probably a reason why, coming back to the discussion with Tom Smith about giving aspirin already to patients with colon cancer, a clinical experiment is still worth doing. Most of the patients will be receiving chemotherapy, and oxaliplatin lowers platelets. So, there are safety issues: does oxaliplatin add bleeding as a complication of the chemotherapy, and is there an interaction between aspirin and the chemotherapy? Third, does oxaliplatin work in the adjuvant setting through reduction of platelets?

To test for an interaction between chemotherapy and aspirin, we have taken not only high-risk stage 2 patients without involved lymph nodes who will be receiving chemotherapy but also stage 2 low-risk patients who will not receive chemotherapy. We have also added a small cohort of patients with metastatic disease in the liver who are undergoing surgical resection with no residual disease to try to get a flavour of the therapeutic effect of aspirin shortly. All patients will be randomised to aspirin 100 mg or placebo daily for five years with an initiation of the treatment within 60 days of the surgery in order to get the potentially important timely effect of aspirin on platelet aggregation.

There are a number of inclusion criteria. Ruth has already discussed the issue of whether aspirin may work specifically in tumour subtypes, now that it is clear that colon cancer is a heterogeneous disease with different driving mutations. We will try to collect all specimens needed for a centralised molecular review, including for instance PI3K mutated tumours.

We have made statistical assumptions. We foresee a 20% reduction in the risk of recurrence. The primary endpoint is disease-free survival at five years, which is conventional in colorectal trials. For this, we need 2,500 patients over all to be recruited in three years. This will be a very difficult trial to conduct because we have believers in aspirin who say that we can no longer do a randomised trial. Some would insist on doing the trial only on patients with PIK3CA mutation. Other oncologists do not believe that aspirin can do anything to prevent cancer metastasis, so will not be persuaded to take part.

The message to the patient will also be a big issue. Informed consent is a big barrier: we must be careful about the mass of information we can give, not to get too many refusals.

### Lung cancer

Lung cancer is the first cause of cancer death worldwide: the death rate, especially in women, is increasing in patients from Eastern Europe and Eastern countries. A meta-analysis taken from figures from many countries shows that aspirin can have a moderate yet significant effect on lung cancer incidence especially evident in case control studies, and slightly less so in cohort studies in which the effect is of borderline significance.

Yet, a 10% reduction by aspirin in the incidence of lung cancer would have profound public health implications. Once again, the most compelling evidence for an effect in lung cancer comes from Prof Rothwell’s data. It shows that low-dose aspirin reduces lung cancer-specific mortality by 30% in a statistically significant way after long follow-up for 20 years. There was no trend with a dose above 75 mg per day, and the effect was particularly evident in adenocarcinoma, the leading type of lung cancer today. The reduction was present in smokers and non-smokers.

The recent US lung cancer screening trial provided compelling evidence of the utility of screening by low-dose computerised tomography. In this large study, CT identified more cancers than were identified on routine chest radiography, but it was associated with lower lung cancer mortality and reduction of overall mortality. There is much discussion on the implementation of these data in Europe and what is clear from such screening programmes is the finding in a large proportion of subjects of undefined and undetermined non-solid nodules.

In screening studies in Milan conducted by Dr Giulia Veronesi, over 10% of the subjects possessed these nodules, called ground-glass opacities. There is some evidence that these GGO may be the precursor lesions of cancer, either of adenocarcinoma or bronchial alveolar carcinoma. Using these opacities as an outcome measure, we conducted a trial of inhaled budesonide (a corticosteroid used for asthma) against placebo that had a negative outcome. However, over the five years of follow-up, the size of the non-solid nodules decreased on budesonide but not on placebo. There was no change in the size of the solid nodules.

We therefore decided to follow this trial with one using aspirin to test if it could shrink the size of GGO in 120 subjects with a baseline of such opacities. The aim was to see if, in principle, aspirin might interfere with the development of the opacities as putative precursor lesions of adenocarcinoma.

Finally, the second biomarker trial is a study of modulation of micro-RNA dysregulation by aspirin. A micro-RNA is a small non-coding RNA molecule found in plants and animals, which functions in transcriptional and post-transcriptional regulation of gene expression. There is some evidence that there are ‘signatures’ in a high-risk population in which circulating miRNA indicates the development of lung cancer and in particular a type that is more aggressive and poses much greater risk. The LUNA study is a Phase II biomarker trial with a few hundred subjects in which will be assessed whether cardioaspirin versus placebo can decrease or revert the progression of the miRNA signature.

To summarise, much is going on in Italy in the medical oncology community. Recruitment will start in 2014 for both the colon cancer study and the ground glass opacity study.

## The future of aspirin—aspirin prophylaxis in the community

### Prof Peter Elwood, Former Director of the Medical Research Council in Wales

In: ‘Securing good health for the whole population’ Wanless stated that health services are unsustainable in their current form unless members of the public are fully engaged and take responsibility for their own health. Representatives of the public agreed in a Citizens’ Jury, accepting that the preservation of health is their own responsibility, and asking for a greater involvement in discussions on public health issues, and in particular in discussions about prophylactic drugs (Elwood & Longley 2010).

The use of aspirin in the reduction of primary vascular disease is, however, controversial, and no regulatory authority has yet approved aspirin for cancer prevention. Nevertheless, almost 40% of people aged over 50 in Wales take aspirin regularly, though almost half take too high a dose. On the other hand, aspirin is underused by patients at seriously increased vascular risk, and it has been estimated that the appropriate use of aspirin in secondary vascular protection could prevent about 700 deaths per year in Wales.

It is to be hoped that if the promotion of low-dose aspirin for healthy, older people becomes acceptable, that it will be promoted within the context of a healthy lifestyle, and never as an alternative to a healthy living. Non-smoking, regular exercise, a low body weight, a healthy diet, and a low alcohol intake together have enormous potential for the reduction of diabetes, vascular disease cancer and even dementia, and information about low-dose aspirin should be given within this context—perhaps even describing it as the sixth healthy behaviour!

Comment about aspirin prophylaxis usually includes advice to subjects: ‘See your doctor first’. At present, this appears to achieve a little, yet the advice has enormous potential for good. Understandably, doctors are risk averse and are highly cautious about recommending a drug that occasionally causes a clinical crisis, yet patients who enquire could easily be asked about symptoms suggestive of gastric pathology and if appropriate, and, if appropriate treated. Blood pressure should be checked and treated if raised. Such actions would be likely to greatly reduce the risk of adverse events.

It is difficult to adequately present all the benefits and risks of long-term aspirin prophylaxis: the risk of a vascular event and the likely reduction by aspirin, the risk of a cancer and the likely reduction by aspirin, the increased risk of a gastrointestinal or a cerebral bleed attributable to aspirin. There is a need, therefore, for a simple visual presentation of all these risks and benefits, and Elwood is working on such a presentation, and this will also include a presentation of the benefits of a healthy lifestyle.

The acceptance of aspirin for the reduction of cancer within public policy and within clinical practice is likely first to be alongside colorectal screening. At present colorectal screening is offered to all adults aged 60–74 years in the UK, and the procedure consists of a request for a faecal sample for testing for blood (an FOB test), followed, if positive, by colonoscopy.

Both preventive measures, screening and low-dose aspirin, are effective and lead to a reduction in cancer incidence. Both procedures also have benefits additional to the reduction of incident cancer: about 10% of subjects with a positive FOB are found to have a neoplastic lesion and a further 10% have ‘high-risk’ adenomatous polyps. Additional benefits from low-dose aspirin include the reduction in vascular disease, and the probable reduction in cancers other than colorectal.

Both procedures, screening and aspirin prophylaxis, also have undesirable side effects. Following colonoscopy, serious bleeding per rectum has been reported to occur in up to six per 1,000 colonoscopies, with a mean rate of 2.5 per 1,000, and perforation of the colon wall occurs in between zero and two (mean 0.74) per 1,000 colonoscopies. Low-dose aspirin is associated with bleeding in 2–3 persons per 1,000 on aspirin in short-term trials, but this rate appears to decrease with time. Cerebral bleeding attributable to aspirin occurs in around two or three per 10,000, and appears to be associated with untreated hypertension.

Serious limitations in screening by colonoscopy are that around 50% of those offered screening refuse the offer, and of those who submit a positive FOB test, around 6% decline colonoscopy (Logan 2012). These raise ethical issues in that an invitation to be screened declares a subject to be at an increased risk of cancer, and it can be argued that subjects who refuse one preventive measure should be offered an alternative even if the alternative is not endorsed by all doctors. The situation with subjects who have a positive FOB is clearly much more urgent.

Several recent reviews of the evidence on colorectal screening and on low-dose aspirin have clearly shown that the addition of aspirin prophylaxis to colorectal screening is both reasonable and cost effective.

Where aspirin prophylaxis will go after all this and what new effects of this simple molecule remain to be discovered is anybody’s guess. Einstein is said to have commented: ‘I never think about the future—it comes soon enough!’ As for aspirin prophylaxis—not soon enough for many of us!

## Figures and Tables

**Table 1:** BHF surveys of UK physicians on reported use of ‘clot busting’ therapy for acute heart attacks before and after the 1988 report of ISIS-2.

Year of survey	Routinely for most patients (%)	Sometimes or as part of a trial (%)	Rarely or never (%)
1987	2	45	53
1989	68	28	3

**Table 2:** Slide Cost-effectiveness ratios—increasing aspirin use from 55% to 75% of eligible populations.

Baseline scenario	£ per CV event avoided	£ per life year gained
*Males*		
25–34	6,113.66	254.74
35–44	1,045.38	55.02
45–54	5.88	0.42
55–64	−336.42	−37.38
65–74	−487.50	−121.87
75+	−561.75	−2,247.00
*Females*		
25–34	564,259.62	22,570.38
35–44	7,016.45	350.82
45–54	461.45	30.76
55–64	−132.60	−13.26
65–74	−267.73	−53.55
75+	−275.44	−220.36
